# 

**Published:** 1904-01-09

**Authors:** 


### CONTENTS.

**Criminal Responsibility of the Insane** - - - 251

**The Importance of Recognising Mild Cases of Diphtheria** - - - - - - - - - - - - 252

**Annotations** - - - - - - - - - - - - - - 253

**Notes and News** - - - - - - - - - - - - - 254

**Hospital Clinics and Medical Progress—**

What We Owe to Experiments on Animals - - - - - - 255

On a Method of Infant-feeding by Home-made Humanised Milk 256

Continuous Local Infection - - - - - - - - - 256

Movable Kidney - - - - - - - - - - - 257

The Nature and Physiological Action of Radium Emanations and Rays - - - - - - - - - - - 257

PROGRESS IN OPHTHALMOLOGY - - - - - - - - 258

PROGRESS IN ABDOMINAL SURGERY - - - - - - - 259

DISEASES OF THE HEART AND CIRCULATION - - - - - 260

**New Appliances and Things Medical** - - - - 261

**The Book World of Medicine and Science** - - 262

**Hospital Administration—**

British Institutions for the Care of the Inebriate.—VII. - - 263

Danish Medical Institutions - - - - - - - 264

Glances at the Hospitals - - - - - - - - 265

**The Student of Medicine** - - - - - - - - 266

*NURSING SECTION.*

**Notes on News from the Nursing World—** The New Queen's Nurses—Belated Honours for Ladysmith Heroines—A Vacant Post at Sydney—Stationary Sisters—Dispensing with the Matron's Signature—Politics and Nursing—The New Matron of the Cancer Hospital—The Congress at Berlin—Hongkong Nursing Institution—British Gynæcological Society—The Nurses of St. Mary's Hospital—A Sister marries a Porter—Nurses as Stewardesses—A Wardswoman as Night Nurse—A Probationer treating a Patient—Brutality by a Drunken Nurse—A Queen's Nurse in an Impossible Position—New Nursing Associations—Nurse and Nursemaid—Short Items . - - - 199-202
**The Nursing Outlook** - - - - - - - - - 203
**Lectures on Ophthalmic Nursing** - - - - - 204
**"The Hospital" Convalescent Fund** - - - 204
**Christmas and New Year Festivities in the Hospitals** - - - - - - - - - - - - 205
**Presentations** - - - - - - - - - - - 207
**Queen Victoria's Jubilee Institute for Nurses** - 208
**Appointments** - - - - - - - - - - - 208
**Echoes from the Outside World** - - - - - 209
**Notes and Queries** - - - - - - - - - 210
**For Reading to the Sick** - - - - - - - - 210

